# Impact of Argon, Nitrogen, and Oxygen Exposure on the Structural and Optoelectrical Properties of Mixed Tin–Lead Halide Perovskites

**DOI:** 10.1021/acsomega.5c00956

**Published:** 2025-06-13

**Authors:** Paula Baltaševičiu̅tė, Rokas Gegevičius, Vidas Pakštas, Arnas Naujokaitis, Vidmantas Gulbinas, Marius Franckevičius

**Affiliations:** 226274 Center for Physical Sciences and Technology (FTMC), Saulėtekio Ave. 3, LT-10257 Vilnius, Lithuania

## Abstract

Mixed tin–lead halide perovskites are considered promising materials for narrow-bandgap photovoltaic applications, particularly in tandem solar cells. However, their practical implementation is hindered by stability issues, especially due to tin oxidation and trap-state formation. In this study, we investigate the impact of argon, nitrogen, and oxygen storage environments on the structural, optical, and electronic properties of mixed tin–lead halide CsFAPb_0.5_Sn_0.5_I_3_ perovskites. Optical absorption, transient photoluminescence (PL), transient photocurrent, and time-delayed collection field (TDCF) measurements reveal the significant role of environmental conditions on carrier dynamics. Carrier trapping over tens of nanoseconds is observed in samples prepared and stored in argon, with a trapping rate increasing several times after exposure to nitrogen (with less than 0.1 ppm of oxygen) and further increasing upon exposure to O_2_. Photocurrent transients also show a fast photocurrent decay component occurring within tens of nanoseconds, independent of the oxygen-created traps. Based on the TDCF measurements, we attribute this fast photocurrent decay component to the spatial traps created by the perovskite boundaries, which reduce the carrier mobility to values below 0.05 cm^2^/V·s, as estimated from transient photocurrent measurements. Our findings highlight the importance of carefully controlling fabrication and storage conditions, often overlooked due to their initially minor impact on device performance, as these conditions critically affect material stability and charge carrier dynamics.

## Graphical abstract

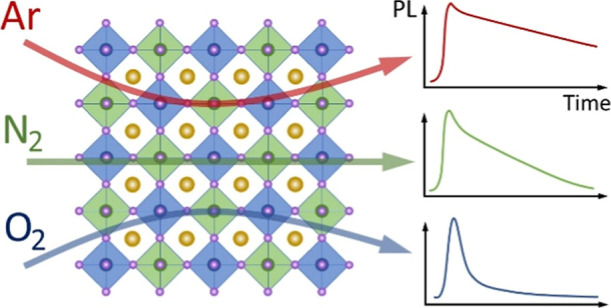

## Introduction

Metal halide perovskite materials became extremely popular in the scientific community due to their attractive optoelectrical properties—high carrier mobility, long carrier diffusion lengths, high absorption coefficients, high defect tolerance, and tunable bandgap energy.^1–4^ This allowed perovskites to be used in various optoelectronic devices, most importantly—solar cells.^5^ However, commonly used Pb-based perovskites have bandgaps in the range of 1.5–1.6 eV, which is slightly above the optimal bandgap for maximum theoretical efficiency in single-junction solar cells (∼1.34 eV). The need for lower bandgap perovskites arises to enable higher theoretical efficiencies in single-junction solar cells while also expanding their potential for tandem applications.^6–8^ Along with this, concerns regarding the toxicity of lead led researchers to study Pb-free or Pb-less perovskite material.^5,7,9^

Alternatives could be tin-, germanium-, or bismuth-based perovskites, but for today, the most promising candidates are mixed tin–lead halide perovskites. These perovskites exhibit optoelectrical and crystallographic properties that are most comparable to those of lead-based perovskites.^10^ Also, mixed tin–lead iodide perovskites have a tunable bandgap between 1.2 and 1.6 eV, which plays a vital role by providing them as a narrow-bandgap absorber layer in all-perovskite tandem solar cells for achieving higher efficiencies compared to single-junction counterparts.^11,12^ In 2024, all-perovskite tandem solar cells where a mixed tin–lead iodide perovskite was used achieved an impressive efficiency of 27.62%.^13^

Environmental factors such as oxygen, nitrogen, and moisture significantly influence the degradation and structural deterioration of mixed tin–lead-based perovskite materials, significantly affecting their long-term stability and performance. The easy oxidation of Sn^2+^ to Sn^4+^ promotes the formation of Sn^2+^ vacancies in the crystal structure, leading to self-p-doping and high densities of trap states.^14^ These trap states are critical in reducing charge carrier mobility and increasing nonradiative recombination losses. Moreover, in mixed tin–lead perovskites, the exposure to air further contributes to the formation of deep trap states, which enhance trap-mediated recombination.^15^ Additionally, the chemical reaction between Sn halides and organic ammonium salts has been demonstrated to contribute to the formation of trap states,^16^ further impeding efficiency and performance of the solar cells.^7^ Under ambient conditions, the oxidation processes are accelerated by exposure to oxygen and moisture, leading to the formation of Sn^4+^ oxides, which act as recombination centers. To suppress the formation of the recombination centers and defects in mixed tin–lead perovskites, various strategies employing reducing agents,^16^ additives,^17^ or solvents^18^ have also been explored.

In this study, we reveal that exposure to oxygen significantly degrades the optical and electronic properties of mixed CsFAPb_0.5_Sn_0.5_I_3_ perovskite thin films with a clear decrease in absorption and the formation of defects. Though the structure remains stable in argon and nitrogen, oxygen exposure leads to tin oxidation, creating defects that reduce carrier mobility. Time-resolved photoluminescence measurements indicate that these defects cause faster carrier recombination and shorter lifetimes, even in the presence of low oxygen levels. Additionally, grain boundary barriers limit carrier mobility, as shown by the fast photocurrent decay observed in all samples. These findings emphasize the crucial role of environmental conditions in the performance and stability of tin–lead perovskite materials.

## Materials and Methods

### Materials

Lead­(II) iodide (PbI_2_, >98.0%) and tin­(II) iodide (SnI_2_, >97.0%) were purchased from TCI Europe N.V. Tin­(II) fluoride (SnF_2_, 99%), *N*,*N*-dimethylformamide (DMF, 99,8%, anhydrous), dimethyl sulfoxide (DMSO, 99.9%, anhydrous), and anisole (99.7%, anhydrous) were purchased from Sigma-Aldrich. Cesium iodide (CsI, 99.9%) was purchased from Thermo Fisher Scientific Inc. Formamidinium iodide (FAI, 99.999%, anhydrous) was purchased from Dyenamo AB.

### Preparation of the Perovskite Precursor Solution

The precursor materials CsI (55 mg), FAI (174 mg), SnI_2_ (225 mg), PbI_2_ (280 mg), and SnF_2_ (10 mg) were weighed in one vial and dissolved in 1 mL of the mixed solvent (*V*_DMF_/*V*_DMSO_ = 2:1). The solution was filtered through 0.45 μm poly­(tetrafluoroethylene) filters before use.

### Film Fabrication

The investigated perovskite films were deposited on microscope slides, which were sequentially cleaned in detergent, distilled water, acetone, and isopropanol in an ultrasonic bath for 15 min each. The cleaned substrates were further treated by oxygen plasma (Diener Femto) for 15 min before the deposition of the materials. The perovskite films were fabricated by an antisolvent approach in an argon-filled glovebox. To maintain consistent fabrication conditions, hydrogen (H_2_) and oxygen (O_2_) levels were carefully controlled to remain below 0.1 ppm (ppm), minimizing contamination or degradation of materials. The two-step spin-coating procedure with 1000 rpm for 10 s and 4000 rpm for 40 s was used. 300 μL anisole was dropped onto the spinning substrates 20 s before the spinning end, followed by 100 °C annealing for 10 min. After the synthesis, the samples were placed into custom-made hermetic measurement chambers equipped with a gas inlet, allowing optical and electrical measurements to be performed under controlled atmospheres. To ensure identical conditions for all measurements, a balloon filled with the corresponding gas (Ar, N_2_, or O_2_) was connected to the chamber inlet, and a controlled gas flow was used during each measurement. This approach enabled precise and consistent control of the sample environment without exposure of the films to ambient air.

Perovskite films used for photocurrent investigations were deposited on interdigitated combs of electrodes (IDEs) using the same deposition conditions as those for films.

### Structural Characterization

The surface morphologies of the films were examined by using a Helios Nanolab 650 scanning electron microscope (SEM). To analyze the phase composition and structural characteristics of the films, X-ray diffraction (XRD) measurements were conducted with a Rigaku SmartLab diffractometer. The crystallite size was determined through the graphical Halder-Wagner method implemented in the PDXL software.

### Optical Characterization

Absorption spectra were measured by using a Jasco V-670 spectrophotometer. Photoluminescence (PL) decay kinetics were recorded with a Hamamatsu C5680 streak camera equipped with a single sweep module (M5677) and coupled to a spectrometer. The excitation source was a femtosecond Yb:KGW oscillator (PHAROS, Light Conversion Ltd.), delivering 80 fs pulses at a wavelength of 1030 nm and a repetition rate of 76 MHz. These pulses were frequency-doubled using a HIRO module (Light Conversion Ltd.) to generate 515 nm light. A pulse picker reduced the repetition rate to 20 kHz. The laser power was attenuated to approximately 200 nW (or 2.5 mW/cm^2^) and focused onto a 100 μm spot on the sample, achieving an excitation energy density of roughly 100 nJ/cm^2^.

### Transient Photocurrent and TDFC Measurements

Transient photocurrent and time-delayed collection field measurements were carried out using an Agilent Technologies DS05054A oscilloscope with a 50 Ω input resistance paired with a Tektronix AFG 3101 function generator. A nanosecond Nd:YAG laser (PL201, Ekspla) delivering 4 ns pulses at a 532 nm wavelength with a repetition rate of 10 Hz was used for sample excitation. The neutral density filters were used to attenuate the intensity of the incident light.

## Results and Discussion

Figure 1 shows the absorption spectra of CsFAPb_0.5_Sn_0.5_I_3_ perovskite thin films that were prepared in an argon atmosphere and subsequently stored in argon, nitrogen, and oxygen environments. The films stored in argon and nitrogen showed no noticeable changes in their absorption spectra after 18 h, indicating that these environments do not induce significant alterations in the electronic properties of the material. In contrast, films exposed to oxygen showed slight changes in their absorption spectra after just 5 h. After 26 h, the absorbance decreased significantly and a slight blue shift of the long-wavelength absorption edge was observed, which clearly indicated significant material degradation, attributed to the high sensitivity of the tin-based perovskite to oxygen. Oxidation of Sn^2+^ to Sn^4+^ is expected to create defects such as tin vacancies and iodide interstitials, which result in p-type doping of a semiconductor.^11,19,20^ These factors lead to a shift of the band edge toward higher energies and a decrease in absorption intensity, as was previously demonstrated by Savill et al.^21,22^

**Figure 1:**
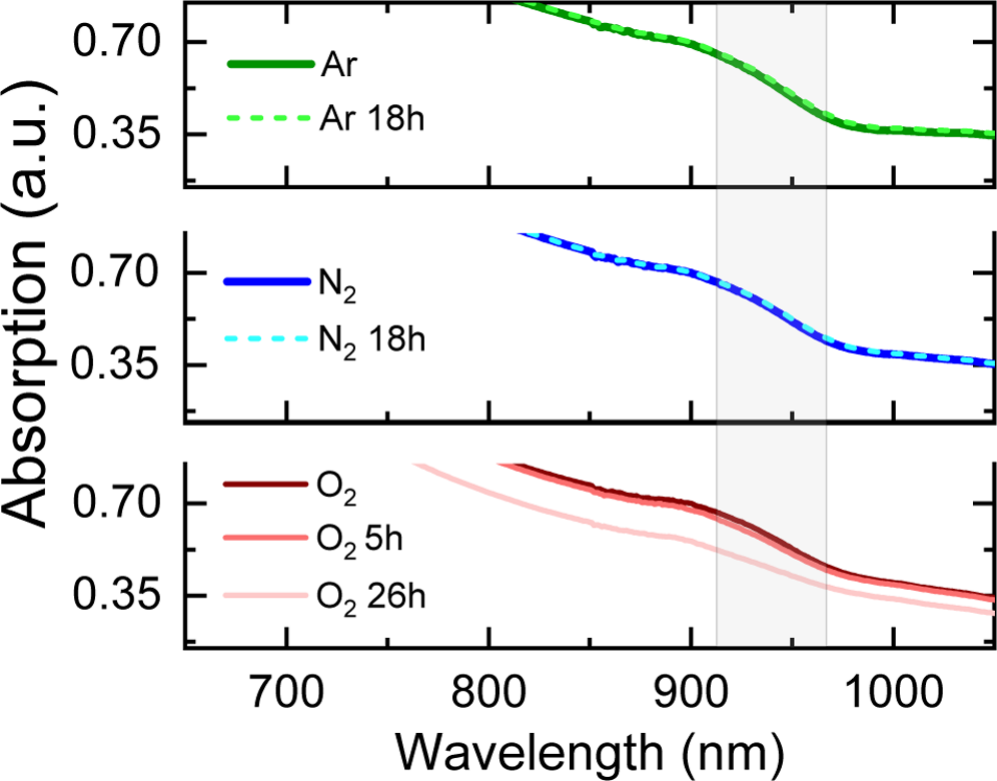
Absorption spectra of CsFAPb_0.5_Sn_0.5_I_3_ perovskite films fabricated under different atmospheric conditions after varying storage times.

To further investigate whether the optical degradation of the CsFAPb_0.5_Sn_0.5_I_3_ perovskite thin films correlates with changes in the crystalline structure, we performed XRD measurements. The results presented in Figure 2 provide information about the composition of crystalline phases and the evolution of structural changes under different environmental conditions. The XRD patterns of the as-prepared mixed lead–tin perovskite films show the characteristic diffraction peaks of the CsFAPb_0.5_Sn_0.5_I_3_ perovskite structure.^23^ Notably, the diffraction patterns of films stored in argon and nitrogen atmospheres remained unchanged after 26 h, indicating that no significant phase transformations or structural degradation occurred during this time. In contrast, the XRD patterns of perovskite films exposed to an oxygen atmosphere for 26 h exhibited a shift of approximately 0.1° toward lower angles. This relatively small shift suggests the presence of lattice distortion or point-defect formation, though no major structural degradation was observed.^15,24^ A similar phenomenon was previously reported for FA_0.75_Cs_0.25_Pb_0.5_Sn_0.5_I_3_ perovskite films stored in air, where minor changes in the XRD patterns were attributed to defect formation rather than structural deterioration of the perovskite lattice.^15^ This defect formation was attributed to the instability of Sn^2+^, which inevitably leads to the formation of tin vacancies, resulting in a high density of hole traps, typically around 10^16^ cm^–3^ in mixed tin–lead perovskites.^12,21^

**Figure 2:**
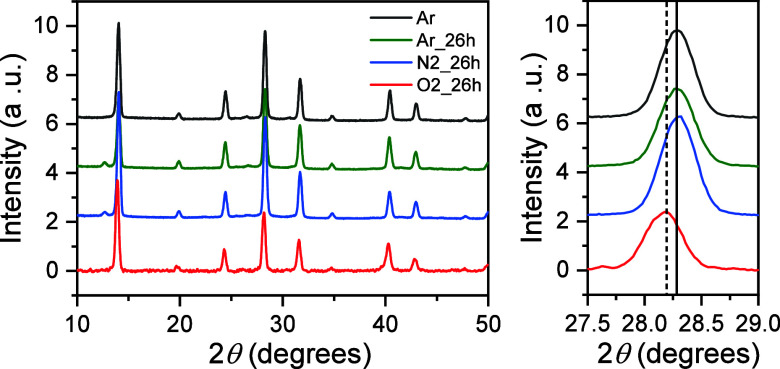
XRD patterns of perovskite films initially fabricated under an argon atmosphere and measured after 26 h of storage in different environments: argon, nitrogen, and oxygen. The right side of the figure shows a zoomed-in view of the (220) diffraction peak over time for each environment, highlighting a slight shift toward lower angles for oxygen-stored films.

To further investigate the changes observed in oxygen, we examined the surface morphology of the perovskite films using SEM. Figure 3 shows the SEM images of freshly prepared films in an argon atmosphere and films exposed to oxygen for 26 h. Both samples show similar morphology, consisting of crystals several hundred nanometers in size, suggesting that the surface structure remains unaffected by oxygen. This further confirms that no surface degradation or structural transformation occurred during the 26 h storage period, in agreement with XRD results. While the observed changes in optical absorption and XRD patterns indicate alterations in the oxygen-exposed films, these changes do not correspond to detectable modifications in the crystalline structure, at least within a 26 h period. Instead, the degradation is likely related to the changes in the surface properties or the electronic characteristics of the material.^15,25^

**Figure 3:**
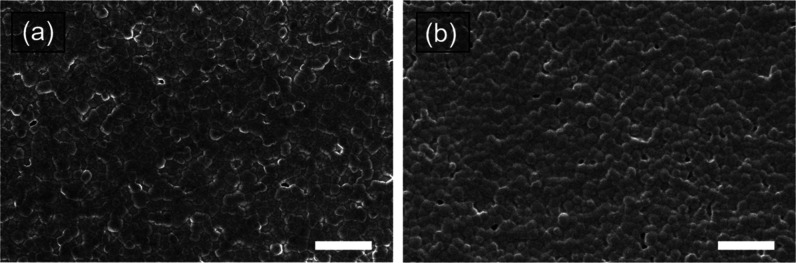
Surface morphology SEM images of the CsFAPb_0.5_Sn_0.5_I_3_ perovskite films fabricated in an argon atmosphere: (a) as-prepared and (b) after being kept in an oxygen atmosphere for 26 h. The scale bars correspond to 2 μm.

To explore electronic processes in the photoexcited mixed perovskite film and their changes upon oxygen exposure, we employed time-resolved characterization techniques. In particular, we focused on elucidating charge carrier dynamics and their correlation with degradation processes in mixed tin–lead halide perovskites. Time-resolved photoluminescence measurements were used to investigate the behavior of photogenerated charge carriers in samples stored in different gas environments (Figure 4). The films were initially prepared and measured in an argon atmosphere. The environment was then switched to nitrogen for 5 min, and measurements were repeated, followed by the same procedure using oxygen. The films kept in an argon atmosphere exhibited long-lived PL decay, with an average lifetime of approximately 58 ns. The obtained lifetime value is similar to those reported for a wide range of Pb–Sn-based perovskite films.^26,27^ Upon exposure to nitrogen for 5 min, the PL lifetime significantly decreased to ∼10 ns. This notable reduction in PL lifetime, despite the use of high-purity nitrogen containing less than 1 ppm of oxygen, suggests that even trace amounts of oxygen can rapidly induce defect formation, accelerating PL decay. Although the types and formation mechanisms of defects differ between lead- and tin-based perovskites,^28,29^ the mixed lead–tin perovskites exhibit intermediate defect formation chemistry and are generally considered to be free of deep traps.^29^ The perovskite films, composed of crystallites several hundred nanometers in size, may still contain a high density of bulk defects. These defects, mainly tin interstitials and iodine vacancies, act as electron traps.^29,30^ Such traps are likely shallow and can localize carriers in nonradiative states, reducing the population of free photogenerated carriers. Consequently, the reduced electron population leads to photoluminescence quenching and enhances Shockley-Read-Hall (SRH) nonradiative recombination, therefore shortening carrier lifetimes.

**Figure 4:**
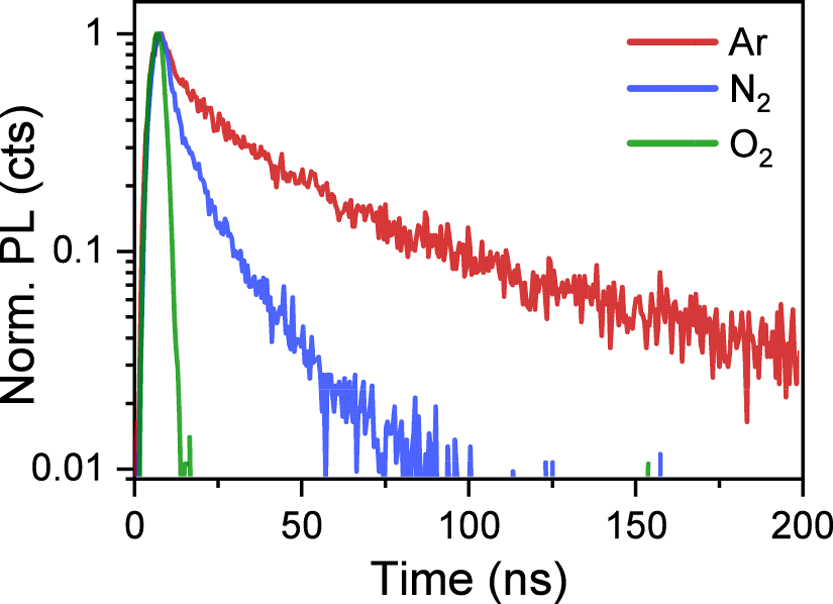
Photoluminescence decay kinetics of mixed lead–tin perovskite films measured under different environmental conditions. All measurements were performed immediately after fabrication, starting in an argon atmosphere.

To gain further insights into the recombination pathways not accessible via PL, we additionally performed transient photocurrent measurements, which provide more information about carrier transport and extraction dynamics. It should be noted that photocurrent transients provide complementary but significantly different information about carrier dynamics compared to PL. PL decays when charge carriers recombine or when at least one type of carrier (electrons or holes) is trapped in nonradiative states. In contrast, photocurrent is determined by the sum of electron and hole concentrations and their mobilities, which changes during time but can remain substantial even when some carriers are trapped. The photocurrent measurements were performed on perovskite films deposited on interdigitated combs of electrodes (IDEs) with a 5 μm interelectrode spacing. The samples were excited using short, about 4 ns, 532 nm laser pulses of varying intensities, while a voltage applied to the IDEs created a lateral electric field to extract charge carriers. The measurement protocol followed the same sequence as in the PL experiments: the films were initially prepared and measured in an argon atmosphere, followed by exposure to nitrogen and finally to oxygen. The transient photocurrent kinetics of films stored in different atmosphere conditions are presented in Figure 5. The kinetics reveals complex dependencies of the photocurrent decays on excitation intensities and applied voltages. We begin our analysis with the intensity-dependent transient photocurrent decays measured at a low applied voltage of 0.1 V (Figure 5a–c), when, as will be discussed below, carrier extraction only slightly alters their concentration. Independent of the sample storage conditions, the photocurrent kinetics at low excitation intensities exhibit two distinct components: a fast decay component with a time constant of about 50–60 ns, and a slower component that persists for nearly a microsecond. As the excitation intensity increases, the fast decay component gradually decreases and finally disappears in films stored in argon and nitrogen atmospheres. This fast decay is attributed to carrier trapping, which reduces their mobility. However, at high excitation intensities, the trap states are rapidly fully populated and no longer influence the carrier mobility. The transient photocurrent decays obtained for the samples stored in Ar and N_2_ are closely similar, which is somewhat surprising given that PL kinetics revealed much faster PL decay in N_2_, indicating increased trap density. For the film stored in oxygen, the shape of the photocurrent kinetics depends only weakly on excitation intensity, consistent with a high trap density that prevents full trap population.

**Figure 5:**
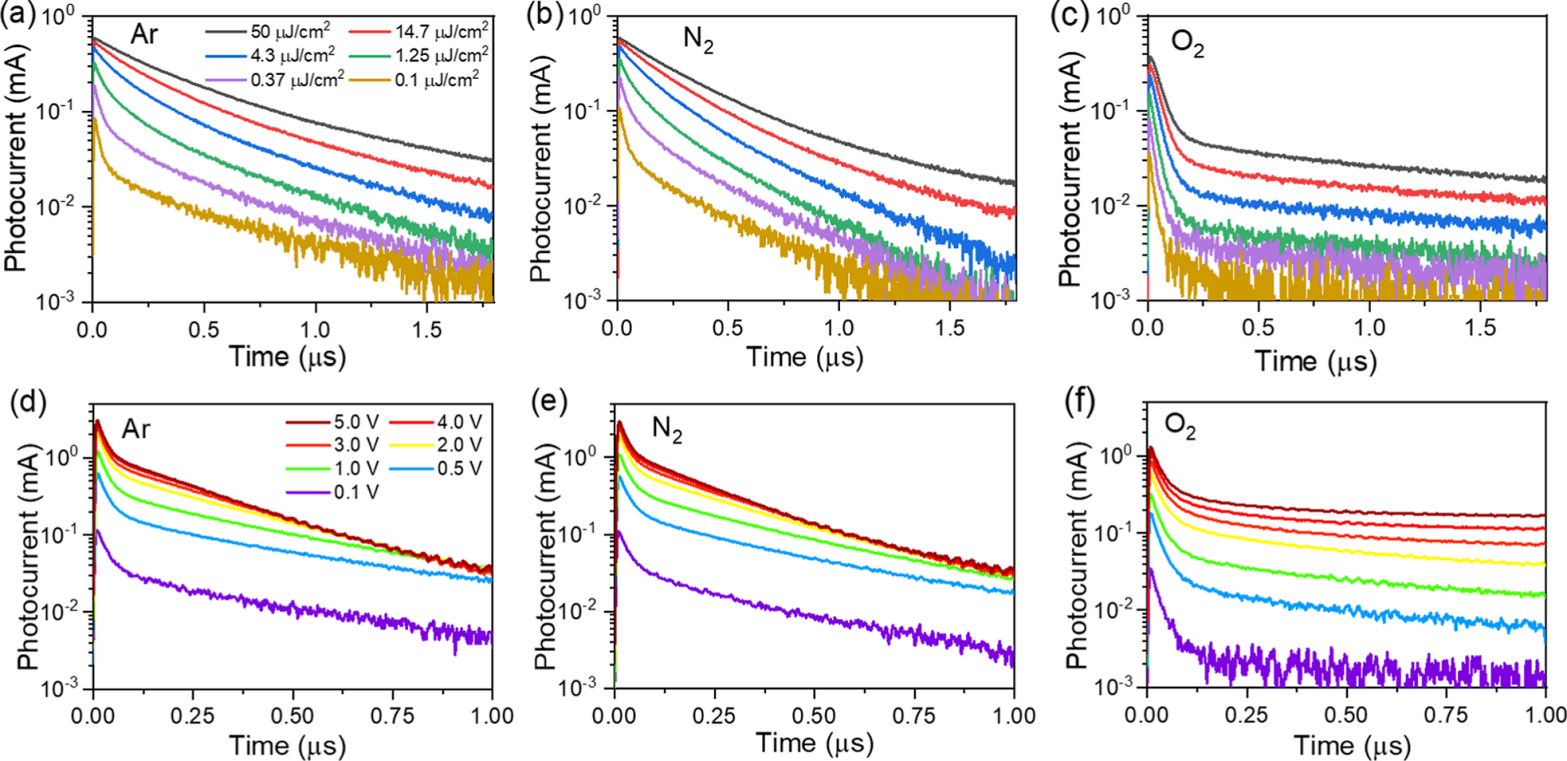
Transient photocurrent decay kinetics of CsFAPb_0.5_Sn_0.5_I_3_ perovskite films on IDEs measured under different gas environments. (a–c) show kinetics measured at varying excitation intensities with an applied voltage of 0.1 V, while (d–f) display kinetics measured at different applied voltages with a constant excitation intensity of 0.1 μJ/cm^2^.

The voltage-dependent transient photocurrent decay kinetics, presented in Figure 5d,e, provide further insights into carrier motion. At low applied voltages (<1 V), the photocurrent transients measured in Ar and N_2_ atmospheres appear nearly identical—their shape does not significantly change with voltage, and their intensities are approximately proportional with the applied voltage. In contrast, at higher applied voltages (>2 V), the decay kinetics reveal an additional fast component, which we attribute to field-driven extraction of photogenerated carriers. Under a sufficiently strong electric field, the charge carriers can efficiently drift across the film and overcome barriers, enabling their collection at the electrodes.

To further analyze the extraction-related features and to estimate carrier mobility, we interpret the observed kinetics using a simplified time-of-flight (TOF) model.^31–33^ Our experimental configuration also corresponds to a TOF method with uniform excitation across the sample along the carrier drift direction. In the ideal case, when electron and hole recombination is absent, and their mobilities are equal and constant, the photocurrent is expected to decay linearly, as illustrated in the inset of Figure 6. Under these conditions, the carrier concentration would decrease two times when carriers drift half distance between the electrodes. The carrier mobility can be expressed as μ = *L* ^2^/(2·*V*·τ_1/2_), where *L* is the distance between electrodes, *V* is the applied voltage, and τ_1/2_ is the time at which the current drops to half its initial value. In our measurements, the photocurrent transients at 0.5 and 0.1 V are nearly identical, suggesting that carrier motion under these conditions is primarily governed by carrier recombination and trapping, rather than by their extraction. Therefore, to obtain carrier extraction-dominated kinetics, we divide kinetics at higher voltages by those measured at 0.5 V. Although this procedure is not strictly mathematically rigorous, the result shown in Figure 6 exhibits almost linear decay for the sample stored in argon, resembling the ideal TOF behavior illustrated in the inset of Figure 6, except for the initial ∼100 ns. Similar kinetics were also obtained for the sample stored in nitrogen. As expected, the slope of the linear decay is approximately proportional to the applied voltage, further validating our approach. Arrows in Figure 6 indicate the time intervals at which the photocurrent decreases by half, yielding carrier mobilities in the range from 3.2 × 10^–2^ cm^2^/V·s to 4.4 × 10^–2^ cm^2^/V·s. These values fall within the broad range reported for mixed Sn–Pb perovskites,^21,34^ depending on film quality, measurement technique, and sample composition. A similar analysis for the sample stored in nitrogen provides comparable mobility values within the limits of experimental accuracy. The initial parts of the kinetics, corresponding approximately to the fast decay component observed in the original kinetics, deviate from the linear regime and indicate that the photocurrent decreases more rapidly at low applied voltages. This deviation is most likely associated with enhanced carrier trapping at low voltages. In contrast, stronger electric field appears to suppress the trapping effect, enabling more efficient carrier extraction.

**Figure 6:**
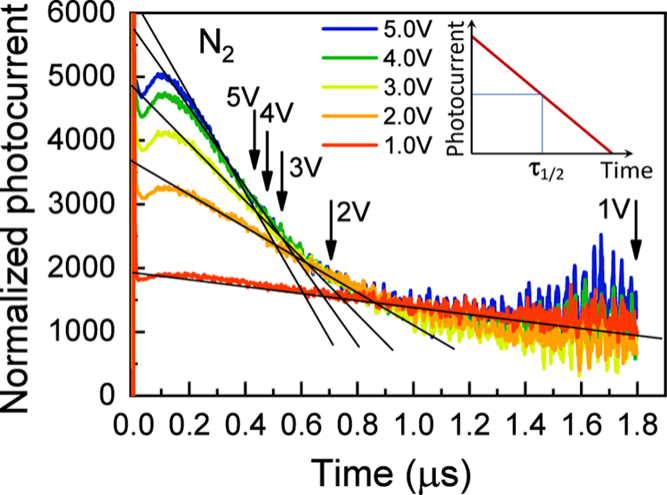
Normalized transient photocurrent kinetics at different applied voltages for the sample in an Ar atmosphere obtained after dividing measured kinetics by kinetics at 0.5 V. Arrows indicate time intervals when the photocurrent decreases twice. The inset shows an ideal time-of-flight kinetics in case of homogeneous along the carrier drift direction excitation.

Although both PL decay and photocurrent transients indicate oxygen-induced formation of additional trap states, some experimental data remain contradictive, such as very similar fast photocurrent decay component remains similar across all samples, despite substantial differences in PL decays. This discrepancy can be attributed to the fact that TRPL and transient photocurrent probe different processes: TRPL is sensitive mainly to radiative recombination of free carriers, whereas the transient photocurrent reflects the transport and extraction of both free and trapped carriers. As a result, the decay kinetics obtained from these two techniques differ significantly. While longer-lived PL components can arise from trap-assisted recombination, our TRPL provides information about fast relaxation processes within tens of nanoseconds that are strongly affected by environmental conditions and directly relevant to charge carrier depopulation in operational devices.

To clarify these discrepancies between PL and transient photocurrent results, we performed time-delayed collection field (TDCF) measurements. In this technique, the samples were excited by short laser pulses, while the voltage extracting carriers were applied after a variable delay. By varying the delay time and measuring the current created by the extraction voltage, we aimed to gain insights into the decay of photogenerated carriers during the interval between optical excitation and carrier extraction. Compared to conventional transient photocurrent measurements, the TDCF technique provides more accurate information about carrier density dynamics that is unaffected by the electric field.

Figure 7 shows the TDCF results obtained for the perovskite sample stored in different gas environments. For these investigations, a low excitation intensity was used, ensuring that the trap filling remained insignificant. The applied extraction voltage of 1 V was also relatively weak, allowing us to neglect any carrier extraction during the initial several microseconds after excitation. We begin our analysis with the sample stored in argon. At zero delay time, when the optical excitation and the extracted voltage pulses are applied simultaneously, we observe a conventional photocurrent kinetics. In the real experiment, the zero-delay time corresponds to the situation where the electrical pulse is applied slightly earlier, by about 10 ns, to compensate the electric field rise time determined by RC of the electric circuit. When the extraction voltage is applied after a delay, we observe a similar kinetics but with a reduced photocurrent amplitude. This reduction is attributed to the decay of the carrier concentration during the waiting period between carrier generation and extraction. The peak photocurrent values as a function of delay time follow almost an exponential decay with a time constant of approximately 700 ns, which we associate with a free carrier lifetime. This behavior is further supported by the normalized *Q*(*t*)/*Q*(0) curves shown in the insets of Figure 7, which reflect the relative decay of the extractable carrier population over time.

**Figure 7:**
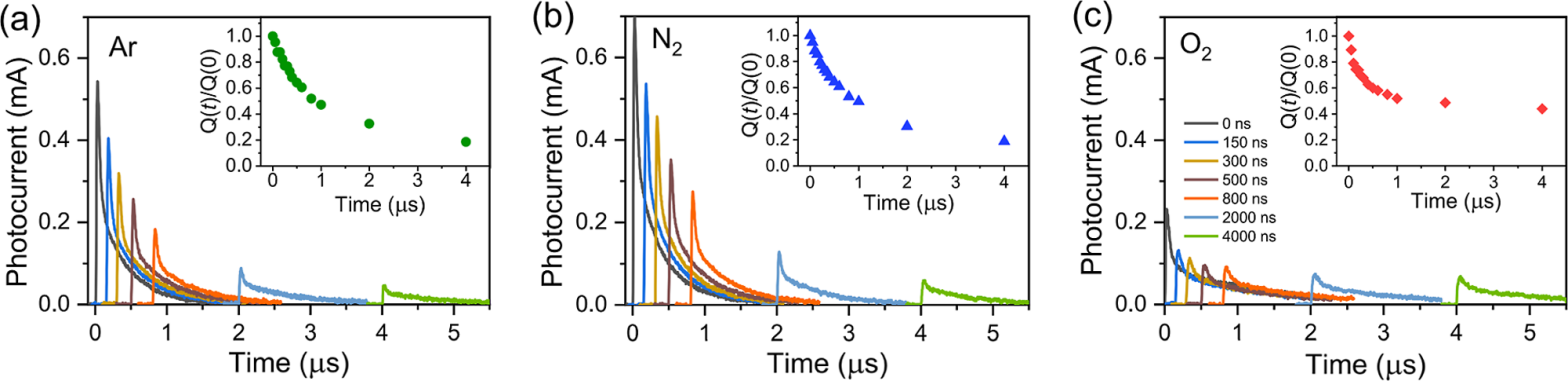
Time-delayed collection field data for CsFAPb_0.5_Sn_0.5_I_3_ perovskite films on interdigitated electrodes (IDE), measured in (a) argon, (b) nitrogen, and (c) oxygen environments. All measurements were performed under a 0 V prebias and a 1 V extraction voltage, with an excitation intensity of 0.1 μJ/cm^2^. Inset plots show the normalized extracted charge *Q*(*t*)/*Q*(0) versus delay time, illustrating the decay of extractable charge carriers.

The TDCF data for the sample stored in nitrogen are nearly identical to those obtained for the argon stored sample. In contrast, the sample stored in oxygen exhibits an additional fast decay component during the initial several hundreds of nanoseconds. At longer times, however, its decay becomes even slower than that for the samples in argon or nitrogen, in agreement with the slower decay observed in conventional photocurrent measurements. These findings highlight additional electronic processes of the mixed Pb–Sn perovskites revealed by the TDCF measurements. Although conventional transient photocurrent also reveals the fast process, however, PL and photocurrent decays show very different properties: (a) the PL decay is significantly faster than the photocurrent decay; (b) the PL decay rates of the three investigated samples are very different, while photocurrent decays are very similar and even photocurrent decay is slightly slower for the sample in O_2_, which shows the fastest PL decay; (c) the PL decays during tens of nanoseconds, while the fast photocurrent component persists even at long delay times.

These differences indicate that the PL and fast photocurrent decay components originate from different processes. Moreover, the process responsible for the PL decay appears to have a little effect on the photocurrent. We speculate that this somewhat unusual behavior may arise if the PL decay is dominated by trapping of one type of charge carriers, which have significantly lower mobility than carrier of another type; therefore, photocurrent is primary determined by the faster carriers that do not experience trapping.

The TDCF measurements provide key insights into the mechanism underlying the fast photocurrent decay component. Notably, this component appears not only immediately after optical excitation but also when the extraction voltage is applied at significantly delayed times after the optical excitation, when all fast carrier relaxation processes are already finished. It leads to the conclusion that the fast photocurrent decay component arises from a rapid decrease in carrier mobility that occurs only when the carriers begin to drift under an applied electric field. Similar mobility dynamics have been observed in MAPI perovskites at low temperatures and were attributed to spatial carrier trapping by potential barriers at grain boundaries.^33^ However, the influence of barriers in MAPI was significant only at low temperatures. In contrast, the barriers in the mixed Pb–Sn perovskites studied here appear to be much higher, as they cause carrier trapping even at room temperature. Furthermore, the observation of this fast photocurrent decay component in all samples independent of the oxygen-created trap state suggests that this is an intrinsic property of the Pb–Sn perovskite system. Our results suggest the scenario in which one type of carrier is rapidly trapped, leading to PL decay, while the other, a nontrapped carrier, exhibits a lifetime of about 700 ns and possesses high initial mobility. These carriers move rapidly only until they reach grain boundaries, which act as barriers for their drift, reducing their mobility to values below 0.05 cm^2^/V·s, as confirmed by our voltage-dependent transient photocurrent analysis.

The high-energy barriers at grain boundaries are probably a result of structural disorder and defect accumulation rather than from phase segregation. These barriers contribute not only to mobility reduction but may also lead to additional PL decay mechanisms, which could arise from the spatial electron and hole separation caused by electrostatic potential variations near the grain boundaries. Although SEM analysis did not reveal any noticeable changes in the grain size across different storage environments, it cannot detect electronic disorder or subtle compositional variations at the grain boundaries that may strongly influence charge carrier transport.

## Conclusions

The optical absorption results reveal that oxygen exposure causes a decrease in absorption and a blue shift in the long-wavelength edge, indicating degradation due to tin oxidation already after 5 h of exposure. XRD analysis shows a slight shift in diffraction peaks toward lower angles for only oxygen-exposed films after 26 h storage, suggesting minor lattice distortion, while films stored in argon and nitrogen remain stable. SEM images confirm that oxygen exposure does not affect the surface morphology. These findings highlight the critical role of environmental conditions, particularly oxygen, in influencing both the optical and structural stability of the tin–lead halide perovskites. The transient photoluminescence and photocurrent kinetics revealed fast relaxation processes over tens of nanoseconds. The PL lifetime variation between the environmental conditions signifies that processes responsible for charge carrier depopulation obviously occur much faster than those detected in absorption and XRD measurements, which require tens of hours to show some noticeable changes, and signify high sensitivity of mixed tin–lead perovskites to even low content of oxygen levels. PL decays kinetics reveal carrier trapping, which is very sensitive even to the very small quantities of oxygen in environmental gases—even less than 0.1 ppm of O_2_ in N_2_ causes several times shortening of the PL relaxation time, while sample exposure to O_2_ shortens the lifetime very significantly. On the other hand, the photocurrent is much less sensitive to the oxygen-formed traps. Significant photocurrent changes are observed only at a high trap concentration created by the sample exposure to oxygen. We attribute this low sensitivity to trapping of carriers which have low mobility and therefore weakly contribute to the photocurrent. However, photocurrent transients also reveal a tens of nanoseconds process, which, according to the TDCF investigation results, should be attributed to the spatial carrier trapping by the grain boundaries reducing their mobility. This process is independent of the trap formation and therefore is an intrinsic property of the Pb–Sn composite perovskite.
